# Tendinopathy VISAs have expired—is it time for outcome renewals?

**DOI:** 10.1007/s00167-021-06596-7

**Published:** 2021-05-10

**Authors:** Vasileios Korakakis, Rod Whiteley, Argyro Kotsifaki, Kristian Thorborg

**Affiliations:** 1 Aspetar Orthopaedic and Sports Medicine Hospital, PO 29222, Doha, Qatar; 2 Department of Orthopaedic Surgery, Sports Orthopedic Research Center – Copenhagen (SORC-C), Amager-Hvidovre Hospital, Faculty of Health Sciences, Copenhagen University, Copenhagen, Denmark

## Introduction

Over 20 years ago, clinical researchers greatly improved our understanding of the multi-faceted nature of the burden of tendinopathy when the first patient-report outcome measure (PROM) in this area was published [19]. The Victorian Institute of Sport Assessment (VISA) questionnaires—Achilles (VISA-A), patellar (VISA-P), hamstring (VISA-H), and greater trochanteric pain syndrome (VISA-G) [2, 5, 15, 19] rate tendinopathy severity from 8 items (question and response/score options) on a single scale from 0 to 100 points, where 100 is the highest and best possible score, indicating no tendinopathy related disability. VISA questionnaires have since dominated the tendinopathy literature as the preferred condition-specific PROMs. This was also recently highlighted as the VISA questionnaires were suggested by the ICON 2019—International Scientific Tendinopathy Symposium Consensus [18] as appropriate and valid measures to capture one of the “core domains” in tendinopathy—*disability*. Ongoing cross-cultural research has already translated and adapted the VISA questionnaires into several languages, with the VISA-A for example, being available in more than 10 languages. The quality of a PROM—not its availability—is fundamental in defining its validity in research and clinical practice [6] and should be based upon its clinimetric properties (i.e., reliability, validity, and responsiveness) [13]. However, in a recent systematic review [8, 9] we have highlighted the limited evidence of the VISA questionnaires’ clinimetric properties and underlined important deviations and short-cuts from methodological recommendations associated with robust and sound development and validation of PROMs. Although, most likely driven by the lack of current and relevant alternatives, the development and replication of VISA study methodology to cover all tendinopathies seem to have introduced bias and affected VISA questionnaires’ validity as a measure of different region-specific tendinopathies.

## Content validity—the patient as the “expert”!

Content validity is the degree to which the content of a PROM is an adequate reflection of the construct to be measured [13]. Patient involvement in the process of item generation and reduction, as well as in the evaluation of relevance (ensuring all items are applicable for assessing tendinopathy), comprehensiveness (warranting all key aspects of the construct are covered) and comprehensibility (items, response options, and instructions are understood by patients as intended) is essential [16]. Input from a diverse group of patients (characteristics, chronicity, severity, activity status) to cover the breadth of the construct “tendinopathy related disability” has been suggested [4]. Unfortunately, the development of the VISA questionnaires did not sufficiently include patients and thus inadequate quality evidence was found for its support [3, 8, 9]. Content validity of the VISA questionnaires was generally based on little or no patient involvement, with the majority (73%) involved being asymptomatic individuals [8]. All VISA development studies [2, 5, 15, 19] were rated as being of “inadequate” quality; however, most of these studies were conducted before the development and publication of the COnsensus-based Standards for the selection of health Measurement INstruments (COSMIN) guidelines in 2012 and it is not surprising that aspects of their methodology would not conform to the standards of the COSMIN initiative. Interestingly, the majority of the published cross-cultural adaptations—despite many being recently published—assessed only comprehensibility, and not cultural adaptation which means that translations of VISA questionnaires have generally not followed existing guidelines [1, 8, 9].

## Structural validity—can VISAs be assumed to measure a single construct?

All VISA development studies [2, 5, 15, 19] used a total score, assuming that all items are a manifestation of the same underlying construct—*tendon-related disability*. Notably, the developers of the VISA-A, VISA-H, and VISA-P [2, 15, 19] suggested that the PROM covers more than one domain (pain, function, and sport activity), while the VISA-G covers disability and activity level [5]. A PROM assessing more than one domain does not conform with unidimensionality [3]. The internal structure of the VISA-A and VISA-P was never evaluated in the development studies [15, 19], but the VISA-H was found to have a 2-factor structure (pain/function and sporting activity) [2], and reported evidence also suggested a 2-factor structure for the VISA-G (pain/function and weight bearing activities) [5]. Recently the Modern Test Theory approach has been developed which includes a collection of statistical models including confirmatory factor analysis, item response theory, and Rasch analysis [3]. This approach is considered the gold standard for validation of patient reported outcomes and their structural validity—and shows that an inconsistent underlying structure for the VISA-A exists violating the assumption that the VISA questionnaires are unidimensional, and thus their computation as a total and single sum score should be avoided [3]. Furthermore, confirmed differential item functioning for sex and across duration of symptoms for VISA-A [3], seem to exist, which means that certain patient groups with similar levels of disability have a different probability of giving a certain response to a particular item.

## Construct validity—concept and hypotheses testing

The extent to which the results of hypotheses testing for construct validity (convergent or divergent) are consistent with the predefined hypotheses based upon underlying constructs and concepts thought to be measured are how a PROM is evaluated and validated [7]. Although our systematic review revealed high-quality evidence for sufficient construct validity of all VISA questionnaires, some general limitations to their construct validity remain. None of the VISA development studies predetermined the expected direction and magnitude of the correlations with the comparator instruments [2, 5, 15, 19], and the majority of the VISA questionnaires validation studies just modelled their methodology based upon the approach of previous studies [8, 9] without any underlying theoretical concept and hypotheses testing. From an in-depth inspection of study methods assessing validity of the VISA questionnaires, 82% of these studies used comparator scales without proven reliability and/or validity, or using non condition- or region-specific PROMs [8, 9]. Hence, the evidence for construct validity of the VISA questionnaires is still in question.

## Current VISAs—expired, time for renewal!

**Fig. 1:**
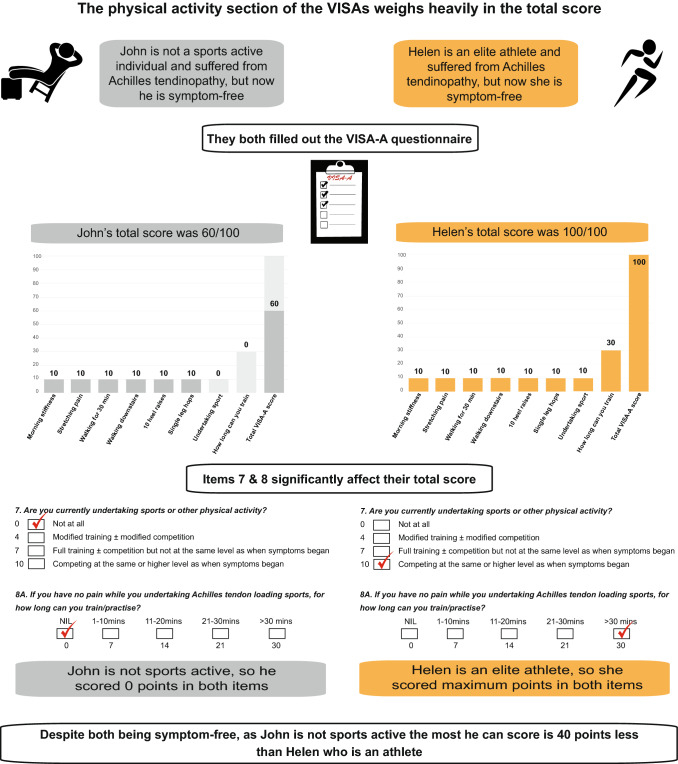
Infographic explaining that the measurement of the tendinopathy construct is problematic with the current scoring structure of the VISA questionnaires

In essence, shortcomings as those highlighted in recent years concerning the VISA-A [3, 11], most likely apply to all VISA questionnaires, as also indicated by our recent systematic reviews in this journal from 2021 [8, 9]. Beyond the obvious evidence and the appraisal of measurement bonafides of VISA from these reviews, the questionnaires incorporate complex items with complicated scoring and thematic ambiguity [3] that needs reconsideration. At face value, the sports/physical activity section in VISA questionnaires is unreasonably heavily weighted in the overall scoring leading to erroneous scoring-based assumptions with regards to disability between active and non-active individuals (Fig. 1) [11]. Moreover, a max score in “item 7” (Are you currently undertaking sport or other physical activity?) in VISA-A, VISA-H, and VISA-P cannot be achieved in people who are not sports active and in VISA-G (Are you currently taking part in regular exercise, physical activity or sport?) in individuals who are not physically active [3]. Given the former, a non-active person’s symptoms may resolve, yet they might only score 50–60 out of 100 [11].

Our knowledge of the multidimensional nature of lower limb tendinopathies has expanded since acknowledging an association of psychological variables and outcomes in tendinopathy [10, 12, 14, 17]. These psychosocial domains should form part of any evaluation of tendinopathy from the patient’s perspective.

The initial description of the VISA [15, 19] took us from oversimplified dichotomous (pain: yes/no, play: yes/no) evaluations of the burden of tendinopathy toward a more detailed and complete description. Over the ensuing 20 years we have learned a lot more about the multi-faceted nature of this disorder’s impact on individuals. For the ICONic status of the VISA questionnaires to remain, updates using robust clinimetric methodology are urgently required. Patient-relevant PROM’s will allow a more complete understanding in our journey towards better outcomes, but before we can continue this path, we need to renew these currently expired VISA questionnaires.

## Abbreviations

PROM: Patient-reported outcome measure
VISA: Victorian Institute of Sport Assessment
VISA-A: Victorian Institute of Sport Assessment Achilles tendinopathy
VISA-G: Victorian Institute of Sport Assessment greater trochanteric pain syndrome
VISA-H: Victorian Institute of Sport Assessment proximal hamstring tendinopathy
VISA-P: Victorian Institute of Sport Assessment patellar tendinopathy
COSMIN: Consensus-based Standards for the selection of health Measurement INstruments
